# Factors Associated with the Underestimation of Manual CPAP Titration Pressure

**DOI:** 10.3390/healthcare11101436

**Published:** 2023-05-15

**Authors:** Po-Yueh Chen, Nguyen-Kieu Viet-Nhi, Yen-Chun Chen, Yi-Lin Kao, Luong Huu Dang, Shih-Han Hung

**Affiliations:** 1 Department of Otolaryngology, Wan Fang Hospital, Taipei Medical University, Taipei 110, Taiwan; poyueh0328@gmail.com (P.-Y.C.); gid5589@gmail.com (Y.-L.K.); 2 Department of Otolaryngology, School of Medicine, College of Medicine, Taipei Medical University, Taipei 110, Taiwan; 3 International Master/Ph.D. Program in Medicine, College of Medicine, Taipei Medical University, Taipei 110, Taiwan; drvietnhi@gmail.com; 4 Department of Otolaryngology, Taipei Medical University Hospital, Taipei 110, Taiwan; cd30617@gmail.com; 5 Graduate Institute of Medical Sciences, College of Medicine, Taipei Medical University, Taipei 110, Taiwan; 6 Department of Otolaryngology, Faculty of Medicine, University of Medicine and Pharmacy at Ho Chi Minh City, Ho Chi Minh City 70000, Vietnam

**Keywords:** COVID-19, obstructive sleep apnea, continuous positive airway pressure, polysomnography, adult

## Abstract

During the SARS-CoV-2 pandemic, the use of in-laboratory positive airway pressure (PAP) titration studies was not routinely suggested. PAP pressure prediction calculations are emerging as alternative methods for the treatment of these patients. The underestimation of PAP titration pressure usually leads to unsatisfactory results for PAP therapy. This study aimed to evaluate the factors associated with the underestimation of PAP titration pressure when using PAP pressure prediction equations. Estimated PAP pressure formulas based on body mass index (BMI) and apnea-hypopnea index (AHI) were chosen to validate the accuracy of equations in the successful prediction of titration pressure. Among 341 adult patients diagnosed with obstructive sleep apnea (OSA) by overnight polysomnography (PSG) and who received overnight PAP titration in order to select a successful pressure, the mean age of the total subjects was 55.4 years old and 78.9% of patients were male. The average BMI and AHI scores were 27.1 ± 4.8 and 37 ± 21.7, respectively. After multivariate stepwise regression analysis, the odds ratio of participants with a pretitration AHI was 1.017 (95% CI: 1.005–1.030). Only the severity of OSA was significantly different between the underestimated group and the adequately assessed group. In conclusion, a high AHI, but not BMI, is associated with an underestimated CPAP titration pressure in adult patients with OSA.

## 1. Introduction

Obstructive sleep apnea (OSA) is a highly prevalent disease worldwide that is associated with systemic consequences, including cardiovascular deficits, neurocognitive function impairment, reduced daytime performance, and impaired driving ability [1,2]. The occurrence of OSA in the general adult population ranges from a 9% to 38% rate [3], and it is more common in men than in women. The prevalence of OSA increases with increasing age and body weight.

The long-term sequelae of OSA include an increased risk of cardiovascular, cerebrovascular, and metabolic syndrome disorders, ultimately leading to premature death if untreated. To ensure optimal long-term outcomes, the assessment and management of OSA should be personalized with the involvement of the appropriate specialists. Continuous positive airway pressure (CPAP) has become the mainstay therapy used to treat adult OSA, especially in moderate to severe diseases. Colin Sulliven first described CPAP therapy as a treatment modality in 1981 [4]. OSA has been thought of as a disorder of anatomical compromise coupled with pharyngeal neuromuscular dysfunction during sleep. Positive airway pressure (PAP) therapy prevents the occurrence of apnea or hypopnea due to upper airway collapse by maintaining a positive upper airway pressure that splints the airway open. Previous systemic reviews have demonstrated that CPAP can reduce the apnea–hypopnea index (AHI), excessive sleepiness, and blood pressure to normal or near-normal levels [5]. PAP use for at least 4 h during one night’s sleep is commonly used to define an acceptable level of compliance clinically. As such, current evidence suggests a dose–response relationship between hours of PAP use and therapeutic effects [4].

In order to achieve a desirable CPAP treatment result, the optimized pressure delivered by the device has to be high enough to splint the airway open, and the procedure to accurately measure the required CPAP pressure is called CPAP titration. Typically, CPAP titration is performed using a CPAP mask fitting and covering the nose or mouth. Then, the air pressure is then delivered through the mask. The delivered pressure gradually increases until the pressure is high enough to support the airway and breathing and sleep become normal. Current manual continuous positive airway pressure (CPAP) titration guidelines recommend starting with a minimum of 4 cmH_2_O and then increasing it by at least 1 cmH_2_O with an interval no shorter than 5 min in order to eliminate obstructive respiratory events. The maximum CPAP is 20 cmH_2_O for patients ≥12 years; if a pressure greater than 15 cmH_2_O is required, they could switch to bilevel-positive airway pressure [6].

During the SARS-CoV-2 pandemic, an in-laboratory positive airway pressure (PAP) study was not suggested unless there was no to minimal risk of SARS-CoV-2 transmission or an extremely urgent case. CPAP titration should be conducted in an airborne infection isolation room with the proper personal protective equipment since it is an aerosol-generating procedure [7]. Facing the dilemma of managing patients diagnosed with OSA by CPAP therapy, telemedicine strategies for CPAP titration and CPAP pressure prediction calculation are emerging as alternative approaches to managing these patients [8,9]. Although CPAP pressure prediction calculations have already been reported, there are variable accuracies of these equations during clinical use [10]. The underestimation of the CPAP titration pressure leads to unsatisfactory results of CPAP therapy and is also related to poor PAP compliance.

In this study, we aimed to evaluate the factors associated with the underestimation of the CPAP titration pressure when using CPAP pressure prediction equations.

## 2. Materials and Methods

### 2.1. Study Population and Design

We retrospectively analyzed the records of all patients 20 years or older who had been diagnosed with OSA using overnight polysomnography (PSG) and received overnight titration at the sleep center of Wan Fang Hospital between 1 January 2016 and 31 December 2020. Patient information, including sex, age, BMI, AHI, and final titration pressure, was collected and analyzed. All of the PSG reports were scored by an experienced sleep technician and reviewed by a sleep specialist blinded to the study groups.

### 2.2. Correlation between the Manual Titration Pressure and the Pressure Predicted by the Formula

The PSG data were analyzed and assessed by a sleep technologist. An apnea event was defined as an airflow reduction ≥90% from baseline for at least 10 s, and a hypopnea event was defined as an airflow reduction ≥30% of baseline for at least 10 s and an accompanying ≥3% oxygen desaturation from baseline and/or arousal ≥3s on an electroencephalogram. This was performed according to the 2012 American Academy of Sleep Medicine recommendations [5].

Previous studies have proposed several titration pressure formulas based on parameters including NC (neck circumference), ODI (oxygen desaturation index), HI (health index), AHI (apnea–hypopnea index), RDI (respiratory disturbance index), smoking in pack years, SSS (snoring severity score), NSAT (nadir saturation), MSAT (mean saturation), nadir SaO_2_ (lowest oxygen saturation) and SpO_2_ (oxygen saturation) [10].

Based on the available information retrievable from the medical record system, two were chosen based on BMI and AHI: 1. Lin et al. 2003 [11] and 2. Choi et al. 2010 [12] validated the accuracy of these formulas in predicting the final titration pressure. The reason for selecting these two equations was based on the fact that these two equations are both generated and validated from the Asian population. The enrolled patients’ estimated pressure was calculated with the following formulas and then compared with the in-laboratory actual titration pressure:

Lin et al. (2003) [11], Taiwan P(eff) = 0.52 + (0.174 × BMI) + (0.042 × AHI); 

Choi et al. (2010) [12], Republic of Korea P(eff) = 0.681 + (0.205 × BMI) + (0.040 × AHI).

### 2.3. Identify Subgroups or Characteristics for Patients with Large Underestimated Titration Pressure 

The characteristics of the participants were identified according to categories of underestimated pressure ≥3 or <3 (optimal pressure > predicted pressure more than 3 cmH_2_O) by Rowley et al. [13].

### 2.4. Statistical Analysis

Data were analyzed using SPSS version 16.0 (IBM, Armonk, New York, NY, USA). As such, continuous variables were presented as the mean ± standard deviation, and categorical variables were presented as the frequency and percentage. Group comparisons of normally distributed quantitative parameters were analyzed using the *t* test and categorical parameters were analyzed with a χ2 test to examine the difference between patient compliance and the patient-related factors of age, sex, BMI, tonsil grade, FTP, Friedman stage, ESS score, overall RDI, NREM RDI, REM RDI, supine RDI, NSAT, and CT90%.

Binary logistic regression analysis was further performed to explore the association between patient compliance and potential factors. Independent variables that were significant in univariate analysis and those reported to affect surgical outcomes were included in the multivariate logistic regression analysis. Significance was defined as a two-sided *p* value of less than 0.05.

A receiver operating characteristic (ROC) curve is a graphical plot that illustrates the diagnostic ability of a binary classifier system, and the area under the curve (AUC) indicates the measure of the ability of a classifier to distinguish between classes. The higher the AUC, the better the performance of the model at distinguishing between the positive and negative classes. All data processing and analysis were performed with R-studio statistical software.

### 2.5. Ethical Approval

The retrospective study of data from patients who were diagnosed with obstructive sleep apnea was approved by the Institutional Review Board of Taipei Medical University (IRB Number: N202201097). We waived the requirement for the informed consent of the patients involved because the study was retrospective and did not violate their rights or adversely affect their welfare.

## 3. Results

After the exclusion of 12 patients with failed CPAP titration, 11 patients needed an optimal pressure greater than 15 cmH_2_O, and 14 patients had an overestimation pressure ≥2, a total of 341 patients with an average age of 55.4 years (range 20 to 92 years) were enrolled in this study. The study included 269 (78.9% men) patients, and the average BMI and AHI values were 27.1 ± 4.8 and 37 ± 21.7, respectively.

According to the different predictive models, the ages of the large underestimation group and the adequate group were 54.9 ± 13.4 and 55.6 ± 14.1 (Lin) and 55.3 ± 13.9 and 55.4 ± 13.8 (Choi), respectively. Men comprised 86.3% and 76.4% (Lin) or 85.5% and 77% (Choi) of the study groups, while BMI was 27.4 ± 4.4 and 26.9 ± 5.1 (Lin) or 27.2 ± 4.3 and 27 ± 5 (Choi), respectively. The pretitration AHI was 42.4 ± 21.2 and 34.3 ± 21.4 (Lin) or 43.5 ± 20.6 and 35.2 ± 21.7 (Choi). PLMS (periodic limb movement during sleep) was 8.6% and 10.7 (Lin) or 9.2% and 10.2% (Choi). There was a lower percentage of large underestimation when using the Choi equation (22.3% vs. 34%). The lower percentage of underestimation of the Choi equation suggests a higher accuracy of the prediction. In an attempt to more accurately identify the predisposing factors leading to the CPAP titration pressure underestimation, the equation was used further in predictor analysis for underestimations. The demographic characteristics of the participants are shown in Table 1 according to categories of underestimated pressure ≥3 or <3 (according to the Choi equation). Only the pretitration AHI showed a significant difference between participants.

Table 2 lists the significant factors and their coefficients; this helps to discriminate patients between the underestimation and adequate groups. The beta coefficient among the parameters was calibrated using stepwise regression analysis. The odds ratio of being in the underestimation group and the adequate group of a participant with a pretitration AHI was 1.017 (95% CI: 1.005–1.030).

**Figure 1:**
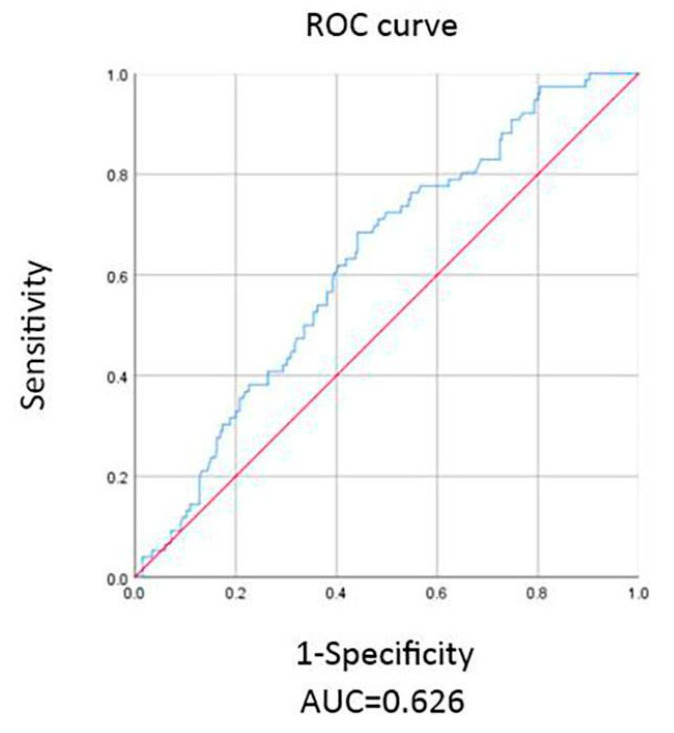
Receiver operation characteristics for large underestimated titration pressure prediction shows the highest area under curve (AUC: 0.626) by Choi estimated equation.

The receiver operating characteristic (ROC) curve is applied in medical diagnostic testing to evaluate the performance of a binary classifier with continuous output, and the area under the ROC curve (AUC) statistic is a common index of model performance. Figure 1 shows that in relying on an AHI cutoff point of 30, our regression model can reach the best diagnostic sensitivity of 71.1% and specificity of 51.7% according to the Youden index [14] when understood via the Choi equation. By using Lin equation, relying on an AHI cutoff point of 32, our regression model can reach the best diagnostic sensitivity of 62.1% and specificity of 43.1% according to the Youden index.

## 4. Discussion

In this study, we demonstrated that a high AHI value, but not a high BMI, is associated with underestimated CPAP titration pressure in patients with OSA. These results provide useful information for physicians and patients, particularly when patients are unable to receive an accurate CPAP titration process, and the pressure must be estimated when setting up the CPAP device. Under these circumstances, as the pressure needed in patients with a high AHI is likely to be underestimated, an alternative protocol of setting up the pressure for CPAP in these patients might be necessary.

CPAP is typically recommended as the gold standard in managing obstructive sleep apnea [15,16]. With the advantage in accuracy, before initiating CPAP therapy, the optimal CPAP pressure is determined by in-laboratory titration. It has been reported that persistent sleepiness after CPAP treatment might result from inadequate titration pressure issues [15]. The current guidelines suggest that either home automatic positive airway pressure (APAP) therapy or in-laboratory CPAP titration should be recommended for patients with OSA compared to no therapy [4,17]. Besides the high accuracy, the benefits of receiving the in-laboratory CPAP titration, in contrast with initiating therapy with APAP at home, are also present in identifying and immediately resolving problems of mask fit or leaks [18].

The disadvantage of manual CPAP titration in the laboratory is that the method is known to be time-consuming and costly [13,16,19]. Additionally, standard in-laboratory titration services might not always be readily available in clinical practices. To lower costs and to initiate CPAP therapy early, split-night studies, autoCPAP titration, home sleep diagnostic studies, unattended autotitrating home CPAP studies, and CPAP prediction formulas have been developed [16]. It has been shown that titration can also be performed using automatic devices in a large number of cases [20]. However, in patients with severe rhinitis or a severe psychiatric or medical disease such as congestive heart failure or significant lung disease, unattended home titration with automatic devices is not recommended; thus, in-laboratory PSG titration is still regarded as the gold standard method of treatment [15]. Moreover, under certain conditions, such as while the SARS-CoV-2 pandemic was ongoing, in-laboratory CPAP titration was considered an aerosol-generating procedure and was not recommended [21]. If CPAP titration during the pandemic appears necessary under certain conditions, the negative screening of COVID-19 patients, the provision of personal protective equipment to protect workers in the laboratory, and a proper cleaning of the environment is required. These requirements increase the cost of the examination, and the possibility of exposure to the virus still exists [7,8,9]. Instead of in-laboratory titrations, many institutes have further shifted this process into telemedicine-based CPAP titration [22]. However, as this telemedicine service is not generalized, some researchers have reported methods for CPAP pressure prediction calculations as being alternatives to the difficulty of performing a titration within the context of the COVID-19 pandemic [9]. Although the use of APAP might be a reasonable alternative, it has been reported that the treatment pressure from APAP tends to be underestimated [23,24,25]. To lower the chance of this underestimation, the starting pressure or minimal pressure based on different prediction equations is commonly preset for APAP therapy [23,25,26].

There have been studies developing prediction equations since Hoffstein et al. published the first equation of this kind using parameters of BMI, NC and AHI. This equation has been further validated by different studies [13,16]. Schiza et al. applied the Hoffstein prediction formula and successfully predicted the optimal CPAP titration pressure in Greek patients. After modifying the equation with smoking history and sex, the successful prediction rate of optimal CPAP pressure within 2 cmH_2_O improved [16]. However, there are also several reports indicating that the formula is somewhat inaccurate. Lee et al. developed their own equation and compared the accuracy of their equation to the Hoffstein equation. The results revealed a significant underestimation by the Hoffstein equation [27]. Tofts et al. reported that both the Hoffstein and Loredo predictive equations underestimate CPAP titration pressures and proposed the use of the Cleveland Clinic Predictor formula as a more accurate option; however, it was only able to accurately predict CPAP in 74% of patients [28]. It seems that instead of further modifying and improving any formula to maximize the accuracy, identifying those who are very unlike to be accurately predicted by the formula, the “outliers”, may be more important and perhaps more effective compared to formula optimization.

In our study, a high AHI but not BMI value was found to be associated with underestimated CPAP titration pressure. This result is supported by a previous study by Rowley et al., who also used the Hoffstein formula to test the accuracy of titration pressure predictions. In their study, the accuracy of the formula was found to be unsatisfactory, with only 30.8% of patients in the successful prediction group. They also found that sex and AHI were associated with successful prediction, and that women and patients with a lower AHI were more prevalent in the successful prediction group. The author further mentioned that the gender distribution in the study was not even, which could contribute to a certain bias [13]. Interestingly, in our study, after adjusting for one of the contributing factors, gender no longer appeared to be a significant contributing factor associated with the underestimation of titration pressure, with the AHI remained statistically significant. The result of our study reveals no gender effect, which is different from the findings of Rowley et al. Interestingly, other similar reports from Asian countries all demonstrated no gender effect [11,12]. This phenomenon might be related to racial differences and implies that the nature and pattern of obstructive sleep apnea might differ between Caucasians and Asians.

A previous study [29] showed that high AHI values at diagnostic night and PLMS should be noted as important indicators of the need to adjust the optimal pressure within the first 5 years of follow-up. According to our study, patients with high AHI were likely to be underestimated for the CPAP titration pressure. With an underestimation of up to 22.3%, patients with an AHI of more than 30 should be frequently and strongly recommended not to skip the in-laboratory titration. While using related prediction formulas still has value in clinical practice, physicians should be reminded about these “outlier” conditions in order to minimize the chance of insufficient treatments being used.

Overall, the underestimation of titration pressure was 22.3%. In the group of AHI above 30, the underestimate of titration pressure was 29.7%. In the group of AHI below 30, the underestimate of titration pressure is 13.8%. For patients with OSA with an AHI of more than 30, CPAP titration pressure should be obtained in the sleep laboratory; for patients with OSA with an AHI of equal or less than 30, the CPAP titration pressure estimation equation can be used to calculate the starting treatment pressure. However, according to a previous study, although these equations might not successfully predict an accurate CPAP titration pressure, the prediction can increase the success rate of CPAP titrations.

There were several limitations of this study. Firstly, this study was retrospective in nature, and thus, only patients receiving a following PSG titration after a confirmed OSA diagnosis were included and this might result in some selection bias. However, as these patient data were retrieved before the COVID-19 outbreak in our country, PSG services were not generally restricted. Second, this study was based on only Chinese patients, and thus, the results of this study might not be suitable for generalization to patients with different baseline profiles, including race, diet and daily activities. Third, as multiple sleep technicians and sleep specialists are involved in scoring the PSG report, some operating errors might result in certain biases in the study measurements. Finally, the true value of identifying patients whose titration pressure might be underestimated remains to be determined, as autotitrating CPAPs are becoming more popular and as the COVID-19 pandemic appears to be under control, and therefore the capacity of the in-laboratory titration is expected to be returning to normal.

It should be noted that a sensitivity of 71.1% and a specificity of 51.7% on an AHI cutoff point of 30 is far from ideal, and additional studies are necessary to select other factors besides the AHI to identify more accurately those who are not suitable for titration pressure prediction for whom in-laboratory titration is necessary. Factors including cephalogram parameters and the influence of medications and underlying conditions were not analyzed in this study. A difference in soft palate length has been proposed as one of the possible factors influencing the accuracy of titration pressure predictions [13]. An elevated AHI could be a consequence of certain anatomical or physiological variations, and more precise predisposing factors must be explored in future studies. Additionally, it would be necessary to further analyze that even when a cutoff point of AHI <30 is applied, many patients will still be whose titration pressure is still underestimated using the equation. The characteristics of these cases might give us some clues in developing a more accurate predicting formula.

Nevertheless, the simple elevated AHI as a predictor of possible underestimation of CPAP titration pressure might still have significant value clinically. This is because the AHI values are very easy to obtain through various sleep diagnostic devices with reasonable accuracy. With the reduced chance of titration pressure underestimation, a positive impact is expected to be observed in the management of OSA patients. Physicians are encouraged to use the result from our study in their clinical practices whenever a titration pressure prediction equation is intended to be used.

Regarding future research directions, a multivariate logistic regression analysis should be explored in those patients with an AHI under 30, but who still possess an underestimated titration pressure, in order to determine the predisposing factors behind these outliers. Besides underestimation, the overestimation of the titration pressure when assessed with the estimation equation may be necessary as well. It is known that in many automated CPAP therapies, the auto-CPAP is set with a minimum pressure in the beginning as the minimum pressure, allowing the pressure not to be dropped too low during the machine’s automatic adjustment processing. While this minimum pressure is commonly set below the actual titration pressure, an overestimated titration pressure might expose the auto-CPAP user to unnecessary strongly positive airway pressure, leading to a higher chance of air leak and discomfort and thus compromising CPAP therapy compliance. Most importantly, as this study is retrospective, it is necessary to conduct a prospective trial to determine whether the selection criteria in this study can be used prospectively to reduce the chance of titration pressure under- and overestimation with the prediction formula. Furthermore, even with a more accurate titration pressure estimation, the actual clinical impact of fine-tuning the titration pressure formula must be validated to see if this will eventually decrease morbidity for these OSA patients.

## 5. Conclusions

In conclusion, patients with AHI ≤ 30 should consider the prediction equation for the starting treatment pressure if conducting a CPAP titration test in the sleep laboratory is difficult. A high AHI, but not a high BMI, is associated with an underestimated CPAP titration pressure in adult patients with OSA. Patients with AHI > 30 should be aware of the underestimation of CPAP pressure and the possibility of undertreatment without the use of formal titration tests at sleep laboratories.

## Figures and Tables

**Table 1:** Characteristics of subjects with underestimated titration pressure ≥3 or not according to estimated pressure by Choi equation.

	ΔPressure (Optimal – Estimated) ≥ 3	ΔPressure < 3	*p* Value
Numbers of subjects (%)	76 (22.3)	265 (77.7)	
Male (%)	65 (85.5)	204 (77)	0.078
Age, mean (SD)	55.3 (13.9)	55.4 (13.8)	0.935
Body mass index, mean (SD)	27.2 (4.3)	27.0 (5.0)	0.735
Pre-AHI, mean (SD)	43.5 (20.6)	35.2 (21.7)	0.003 *
PLMI ≥ 15 (%)	7 (9.2)	27 (10.2)	0.803

* *p* < 0.05.

**Table 2:** Clinical predictors of large underestimated titration pressure using estimated equation after adjustment for age, sex, BMI and PLMS.

	Coefficient	Odds Ratio	95% CI	*p* Value
Constant	−1.799	0.165		0.112
Male	0.440	1.553	2.76–3.18	0.228
Age	−0.003	0.997	0.98–1.02	0.789
BMI	−0.012	0.988	0.93–1.05	0.685
Pre-AHI	0.017	1.017	1.01–1.03	0.007 *
PLMI ≥ 15	0.100	1.106	0.45–2.73	0.827

* *p* < 0.05.

## Data Availability

Data sharing not applicable.
